# Outcomes of a 2-year treat-and-extend regimen with aflibercept for diabetic macular edema

**DOI:** 10.1038/s41598-021-83811-y

**Published:** 2021-02-24

**Authors:** Takao Hirano, Yuichi Toriyama, Yoshihiro Takamura, Masahiko Sugimoto, Taiji Nagaoka, Yoshimi Sugiura, Fumiki Okamoto, Michiyuki Saito, Kousuke Noda, Shigeo Yoshida, Akihiro Ishibazawa, Osamu Sawada, Toshinori Murata

**Affiliations:** 1 Department of Ophthalmology, Shinshu University School of Medicine, 3-1-1 Asahi, Matsumoto, Nagano 390-8621 Japan; 2 Department of Ophthalmology, Faculty of Medical Sciences, University of Fukui, Eiheiji, Yoshida, Fukui 910-1193 Japan; 3 Department of Ophthalmology, Mie University Graduate School of Medicine, 2-174, Edobashi, Tsu, Mie 514-8507 Japan; 4 Department of Ophthalmology, Nihon University Itabashi Hospital, 30-1 Ooyaguchikami-machi, Itabashi, Tokyo 173-8610 Japan; 5 Department of Ophthalmology, University of Tsukuba Faculty of Medicine, 2-1-1 Amakubo, Tsukuba, Ibaraki 305-8576 Japan; 6 Department of Ophthalmology, Faculty of Medicine and Graduate School of Medicine, Hokkaido University, Kita 15, Nishi 7, Kita, Sapporo 060-8638 Japan; 7 Department of Ophthalmology, Kurume University School of Medicine, 67 Asahi, Kurume, Fukuoka 830-0011 Japan; 8 Department of Ophthalmology, Asahikawa Medical University, 2-1-1 Midorigaokahigasi, Asahikawa, 078-8510 Japan; 9 Department of Ophthalmology, Shiga University of Medical Science, Seta Tsukinowa, Otsu, Shiga 520-2192 Japan

**Keywords:** Medical research, Eye diseases

## Abstract

This prospective, open-label, single-arm, non-randomized clinical trial, assessed the efficacy of a 2-year treat-and-extend (T&E) regimen involving intravitreal aflibercept injection (IAI), with the longest treatment interval set to 16 weeks, and adjunct focal/grid laser in diabetic macula edema (DME) patients. We examined 40 eyes (40 adults) with fovea-involving DME from 8 Japanese centers between April 2015 and February 2017. Participants received IAI with an induction period featuring monthly injections and a subsequent T&E period featuring 8–16-week injection interval, adjusted based on optical coherence tomography findings. The primary endpoints were mean changes in the best-corrected visual acuity (BCVA) and central subfield macular thickness (CST) from baseline. Thirty patients (75%) completed the 2-year follow-up. The mean BCVA and CST changed from 60.5 ± 15.6 letters and 499.2 ± 105.6 µm at baseline to 66.6 ± 17.1 letters (P = 0.217) and 315.2 ± 79.0 µm (P < 0.001), respectively, after 2 years. The treatment interval was extended to 12 and 16 weeks in 6.7% and 66.7% of patients, respectively, at the end of 2 years. The T&E aflibercept regimen with the longest treatment interval set to 16 weeks, with adjunct focal/grid laser may be a rational 2-year treatment strategy for DME.

## Introduction

Diabetic macula edema (DME) is a major cause of vision loss in the working population^1^. Focal/grid laser was a common first-line treatment for DME until the advent of anti-vascular endothelial growth factor (VEGF) therapy^2^. The Diabetic Retinopathy Clinical Research network reported that intra-vitreous injections of anti-VEGF agents demonstrated much better efficacy in the resolution of macular edema and improvements in vision than the conventional focal/grid laser^3^. Pivotal randomized clinical trials (RCTs) that used bevacizumab^4^, ranibizumab^5^, and aflibercept^6^ also reported that anti-VEGF therapy carries better efficacy than focal/grid laser; therefore, anti-VEGF therapy is the first line treatment for DME now. However, while the excellent effects of anti-VEGF therapy have been widely accepted, we also have found that the proposed regimens by the aforementioned RCTs in patients with DME^7^ or age-related macular degeneration^8^ is not always possible in real-world settings^9^. A majority of the RCTs employed fixed-dosing regimens, such as monthly^5,10^ or bimonthly^11,12^ injections, which requires frequent hospital visits. Most RCTs included a 2–5-year protocol^5,11^, and anti-VEGF therapy in DME usually lasts for at least several years. The financial costs of multiple anti-VEGF injections are sometimes not affordable for patients with DME, especially, after the second or third year, which can lead to deterioration in vision^13^. Consequently, various modified treatment regimens have been evaluated to reduce the number of injections and follow-up visits while maintaining the therapeutic effects. Combined therapy with anti-VEGF injections and other conventional treatments, such as focal/grid laser^14,15^, and corticosteroids^16^ has demonstrated modest effects in reducing the required number of injections compared with monotherapy. In an attempt to reduce the number of injections than fixed dosing, 2 regimens were developed—a pro re nata (PRN) regimen and a treat-and-extend (T&E) regimen^17^.

In the PRN regimen, the number of anti-VEGF injections may be reduced as these injections are only administered in cases of recurrence of macular edema. However, the patients are required to regularly visit the hospitals, usually monthly, which is still a substantial burden for patients of working age. Furthermore, the vision in these patients tends to gradually deteriorate over time because periodic recurrences of macular edema between the hospital visits gradually sum up over the long run. The T&E regimen was developed to avoid such periodic recurrences of macular edema with the use of regular anti-VEGF injections. At every visit, the resolution of macular edema is confirmed using optical coherence tomography (OCT) and the intervals are gradually extended by 2 weeks or 1 month^11,17^. Therefore, T&E regimens can reduce both the number of injections and hospital visits while maintaining remission of macular edema.

The T&E method has been used with both ranibizumab^18^ and aflibercept^19^ in the treatment of age-related macular degeneration. Good therapeutic efficacy of the T&E regimen with ranibizumab in DME has also been reported^20^. However, there are only a few reports regarding the clinical efficacy of T&E protocols involving aflibercept injection for DME^21,22^, and the treatment interval in those reports was set to a maximum of 12 weeks. The ALTAIR study that examined a T&E regimen with aflibercept for AMD reported that up to week 96, the injection interval was extended to 16 weeks for 46.3% patients in the group with 4-week adjustments^23^. Additionally, the vitreous half-life of aflibercept is longer than that of ranibizumab^24^. Therefore, we hypothesized that T&E regimens with aflibercept may extend the injection intervals to longer than 12 weeks, which should further reduce the number of injections.

Consequently, we investigated the efficacy of a T&E regimen involving aflibercept injections with the maximum treatment interval set to 16 weeks over the second year and summarized the results of the 2-year study in this report.

## Methods

### Study design and patients

This prospective, open-label, investigator-initiated, multicenter, single-arm clinical study included 40 eyes of 40 patients with DME. Patients were enrolled from 8 centers in Japan between April 1, 2015, and February 28, 2017. The study was approved by the ethics committee of the Shinshu University School of Medicine (approval number: 3111), and it was performed according to the tenets of the Declaration of Helsinki. Written informed consent was obtained from all patients. The study was registered with the University Hospital Medical Information Network (22/03/2015, identifier: UMIN000016867). The eligibility criteria conformed to those of the VISTA and VIVID studies^6,11^, which reported the efficacy and safety of IAIs (Eylea, Regeneron, Tarrytown, NY, USA and Bayer HealthCare, Berlin, Germany, respectively) in DME; however, the age criterion was ≥ 20 years in this study without upper limit on visual acuity (VA) (Supplementary Table S1). The main inclusion criteria were: (1) age ≥ 20 years and type 1 or type 2 diabetes mellitus; (2) patients with fovea-involving DME, defined as a 300-µm CST measured as the mean retinal thickness in the central 1-mm diameter circle using spectral domain (SD)-OCT (Cirrus OCT; Carl Zeiss Meditec, Inc., Dublin, CA, USA); (3) patients with DME and visual impairment; and (4) patients with best-corrected visual acuity (BCVA) score ≥ 24 letters based on ETDRS VA charts. Data collected at baseline included age, sex, blood hemoglobin A1c level, duration of diabetes mellitus, blood hemoglobin level, diabetic retinopathy severity, serum creatinine level, and diastolic and systolic blood pressures. At baseline and during the follow-up, the patients underwent complete ophthalmic examinations that included ETDRS VA testing, intraocular pressure measurement, slit-lamp biomicroscopy, indirect ophthalmoscopy, fundus photography, and SD-OCT. Fluorescein angiography (FA) using confocal scanning laser ophthalmoscopy (HRA-2; Heidelberg Engineering, Inc., Dossenheim, Germany) was performed at baseline to detect leaking microaneurysms and capillary dropout areas as the targets for focal/grid photocoagulation. Although most examinations were performed using the aforementioned methods, comparable methods were used at some centers. Since this was a single-arm study, we compared the results to those of the VISTA and VIVID studies^6,11^. The independent study control center was managed, and all data were collected by a contract research organization (Satt Co., Ltd., Tokyo, Japan).

### Treatment

The treatment protocol consisted of 2 phases: the induction phase and the T&E phase. Eligible patients received 2 mg IAI at the initial visit and each subsequent visit. The 16-week period after the first IAI was considered the induction phase, during which monthly IAIs were administered for 5 months. One week after the first IAI, short-pulse focal/grid photocoagulation was performed on the FA findings according to previous reports^15^ as follows: Focal burns were delivered to leaking microaneurysms (MAs) at the settings of: (1) spot size of 50 µm; (2) duration of 0.02–0.03 s.; and (3) power ranging 100–250 mW to achieve a mild whitening of MAs. Grid laser photocoagulation was delivered to the thickened retinal areas with capillary nonperfusion or diffuse leakage within the vascular arcades at the settings of: (1) spot size of 50 µm; (2) duration of 0.03 s.; and (3) power ranging from 100 to 250 mW to achieve vaguely visible laser burns. Thereafter, focal/grid photocoagulation was performed ≥ 4 weeks after the previous session of focal/grid photocoagulation. If a participant did not meet any of the re-injection criteria, which included (1) increase in CST > 150 μm from the nadir value on OCT and (2) new or persistent cystic retinal changes or subretinal fluid identified on OCT or persistent diffuse edema with CST ≥ 350 μm 8 weeks after the first injection, the disease was considered to be stable, and the patient continued into the T&E phase. As an exception, if a participant met any of the aforementioned re-injection criteria after the fourth IAI, the disease was considered to have recurred, and a fifth IAI was administered 4 weeks (not 8 weeks) after the fourth injection, which is the shortest interval for visits during the T&E phase. The subsequent study procedures were performed similar those in the T&E phase. Sixteen weeks after the first IAI, participants received IAIs at 8-week intervals. For those who did not satisfy any of the re-injection criteria at a visit, the intervals were extended from 8 to 16 weeks (maximum) in 4-week increments. If any of the re-injection criteria were satisfied at a subsequent visit, the intervals were shortened again to 8 weeks. If the re-injection criteria were not satisfied at a subsequent visit, the intervals were extended again to 16 weeks (maximum) in 4-week increments.

### Outcome measures

The primary endpoints of this study were the mean BCVA and CST changes from baseline at 2 years. The secondary endpoints were the mean number of IAIs, treatment interval distributions at 2 years, and ocular and systemic adverse events over the two-year period. Additional outcomes included the mean largest BCVA and CST improvements from baseline over 2 years.

### Statistical analysis

Non-inferiority analyses were completed for the full analysis set (FAS) and the per-protocol set (PPS). The FAS population included all patients who received at least 1 dose of the study medication. The last observation carried forward method was used to impute any missing values for the analysis in the FAS population. Changes in each test value after the treatment were tabulated every 4 weeks, and changes from the baseline at each time point were evaluated using repeated measures analysis of variance (ANOVA) and multiple comparison test (Dunnett’s method) between the baseline and each time point. Continuous variables are expressed as mean ± standard deviation. Categorical variables are expressed as numbers and percentages. The significance level in this analysis was 5% on both sides. All analyses were performed using SPSS Statistics v23.0 (IBM Corp., Armonk, NY, USA).

## Results

### Baseline characteristics and patient demographics

Overall, 42 patients were enrolled across 8 clinical centers. Of them, 2 were not included in the efficacy analysis since they did not receive IAIs. The remaining 40 patients constituted the FAS population; of them, 30 (75%) completed the 2-year follow-up and adhered to the protocol. These 30 patients comprised the PPS population—the efficacy population for the analyses. The baseline characteristics and patient demographics of the FAS and PPS populations are summarized in Table 1 and Supplementary Table S2, respectively.

**Table 1:** Participant demographics and baseline characteristics.

Characteristic	Participants (n = 40)
Age, years	66.0 (9.1)
Female, n (%)	15 (37.5)
HbA1c, %	7.2 (1.0)
Duration of diabetes, years	11.8 (8.3)
Central retinal thickness, μm	501.9 (109.4)
Previous treatment for DME, n (direct or grid laser, anti-VEGF, naïve)	11/9/24
DME morphological subtypes, n (DRT alone, DRT + CME, DRT + SRD, DRT + CME + SRD)	2/25/5/8
DR severity, n (mild/moderate/severe NPDR/PDR)	4/17/13/6
Cr, mg/dl	0.95 (0.52)
Hb, g/dl	13.5 (1.5)
**Blood pressure, mmHg**	
Systolic	131.0 (15.4)
Diastolic	75.8 (11.1)

Data are presented as mean (standard deviation).

*CME* cystoid macular edema, *Cr* creatinine, *DME* diabetic macular edema, *DR* diabetic retinopathy, *DRT* diffused retinal thickening, *Hb* Hemoglobin, *HbA1c* Hemoglobin A1c, *NPDR* non-proliferative diabetic retinopathy, *PDR* proliferative diabetic retinopathy, *SRD* serous retinal detachment, *VEGF* vascular endothelial growth factor.

### VA outcome

**Figure 1:**
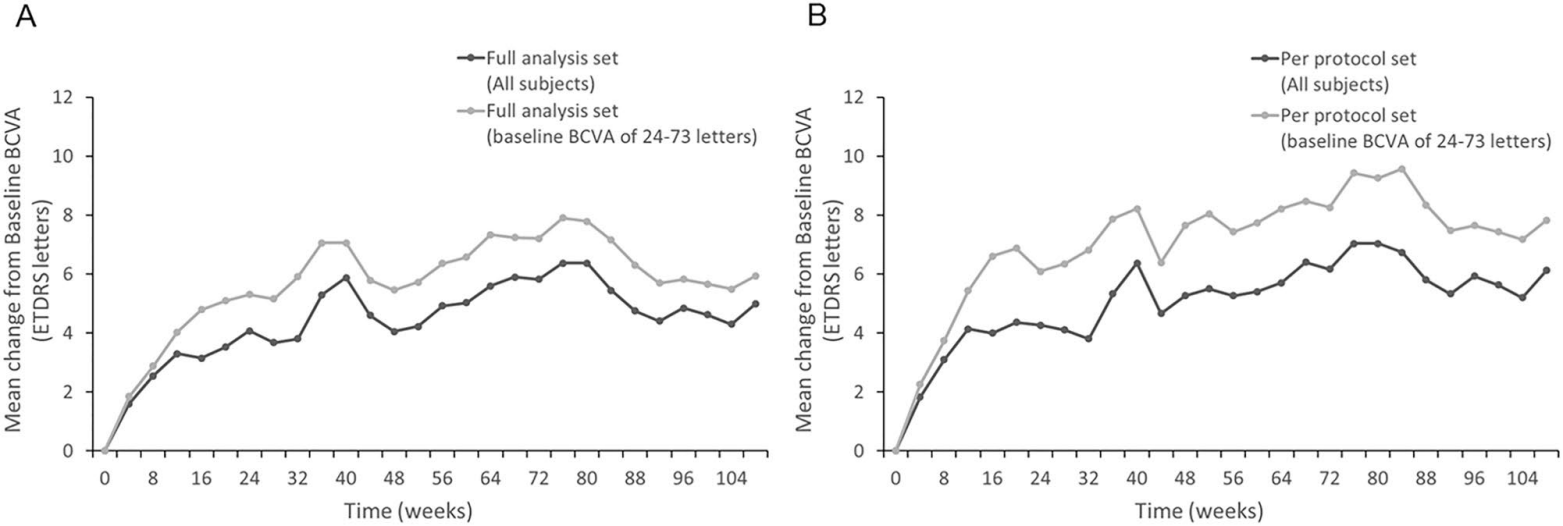
Change in the mean best-corrected visual acuity. Change in the mean best-corrected visual acuity (BCVA; Early Treatment Diabetic Retinopathy Study letters) in (**A**) full analysis set (all participants and those with baseline BCVA of 24–73 letters) and (**B**) per-protocol set (all participants and those with baseline BCVA of 24–73 letters). Missing values are imputed using the “Last Observation Carried Forward” method.

**Figure 2:**
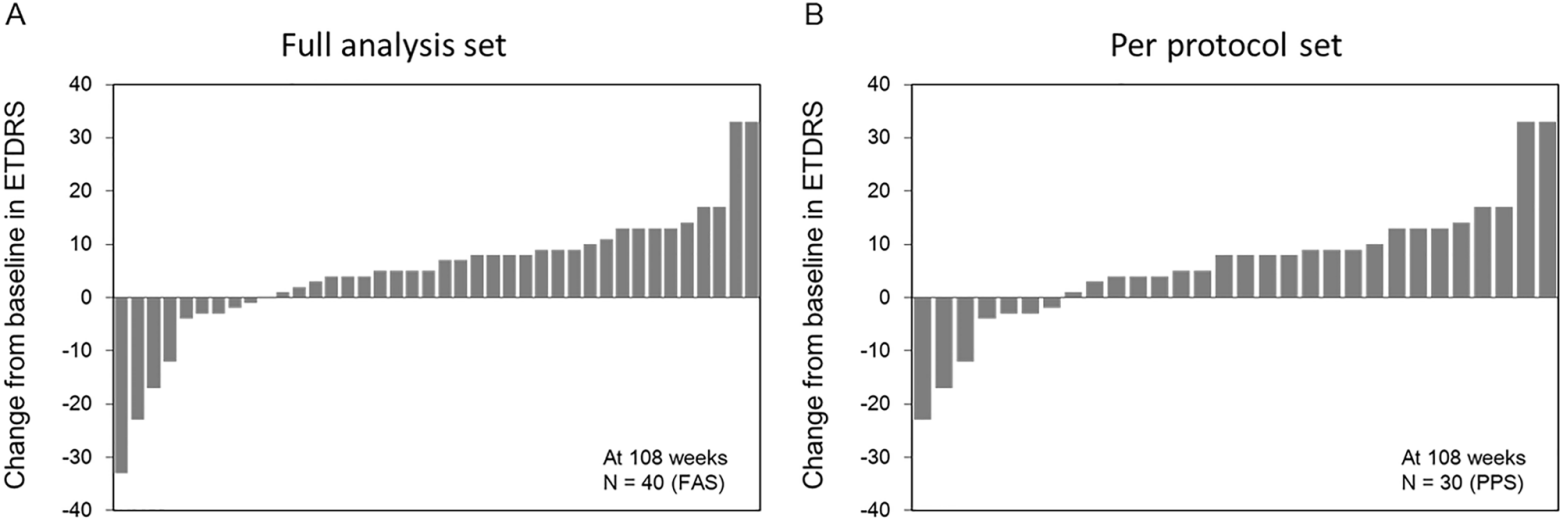
Graphs illustrating individual changes in best corrected visual acuity letter score (Early Treatment Diabetic Retinopathy Study: ETDRS). Each bar corresponds to an individual patient.

In the FAS participants (n = 40), the mean ETDRS BCVA improved by 5.0 ± 12.1 letters, (baseline: 59.9 ± 14.4 letters; at 2 years: 64.9 ± 16.8 letters; P = 0.340, Fig. 1A). In 33 participants with baseline BCVA of 24–73 letters, the same inclusion criteria as those of the VISTA and VIVID studies, the mean ETDRS BCVA improved by 5.9 ± 12.8 letters (baseline: 56.4 ± 13.4 letters; at 2 years: 62.3 ± 17.0 letters; P = 0.316, Fig. 1A). Similarly, in the PPS population (n = 30), the mean ETDRS BCVA improved by 6.1 ± 11.8 letters (baseline: 60.5 ± 15.6 letters; at 2 years: 66.6 ± 17.1 letters; P = 0.217, Fig. 1B). In 23 participants with baseline BCVA of 24–73 letters, the mean ETDRS BCVA improved by 7.8 ± 12.5 letters (baseline: 55.7 ± 14.7 letters; at 2 years: 63.5 ± 18.0 letters; P = 0.177, Fig. 1B). Waterfall plots for the changes in BCVA for individual eyes indicated that few patients experienced any loss of vision (Fig. 2).

### Anatomical outcomes

**Figure 3:**
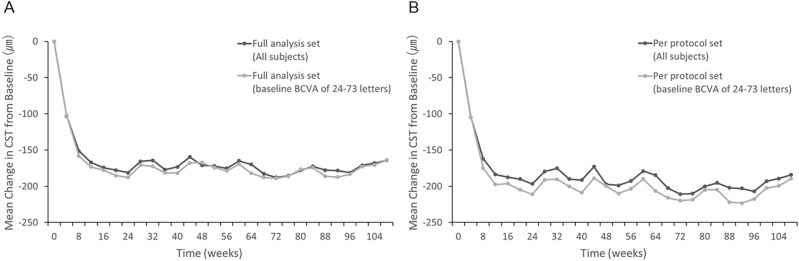
Change in the mean central macular thickness. Change in the mean central macular thickness (CST) in (**A**) full analysis set (all participants and those with baseline BCVA of 24–73 letters) and (**B**) per-protocol set (all participants and those with baseline BCVA of 24–73 letters). Missing values are imputed using the “last observation carried forward” method.

In the FAS population (n = 40), the mean CST decreased by 164.1 ± 119.4 µm (baseline: 501.9 ± 109.4 µm; at 2 years: 337.8 ± 86.8 µm; P < 0.001, Fig. 3A). In the 33 participants with baseline BCVA of 24–73 letters, the mean CST decreased by 163.7 ± 120.6 µm (baseline: 505.3 ± 114.6 µm; at 2 years: 341.6 ± 89.3 µm; P < 0.001, Fig. 3A). Similarly, in the PPS population (n = 30), the mean CST decreased by 184.1 ± 122.3 µm (baseline: 499.2 ± 105.6 µm; at 2 years: 315.2 ± 79.0 µm; P < 0.001, Fig. 3B). In the 23 participants with baseline BCVA of 24–73 letters, the mean CST decreased by 189.5 ± 124.6 µm (baseline: 503.3 ± 112.3 µm; at 2 years: 313.7 ± 81.2; P < 0.001, Fig. 3B).

### Number of treatments and length of intervals

**Figure 4:**
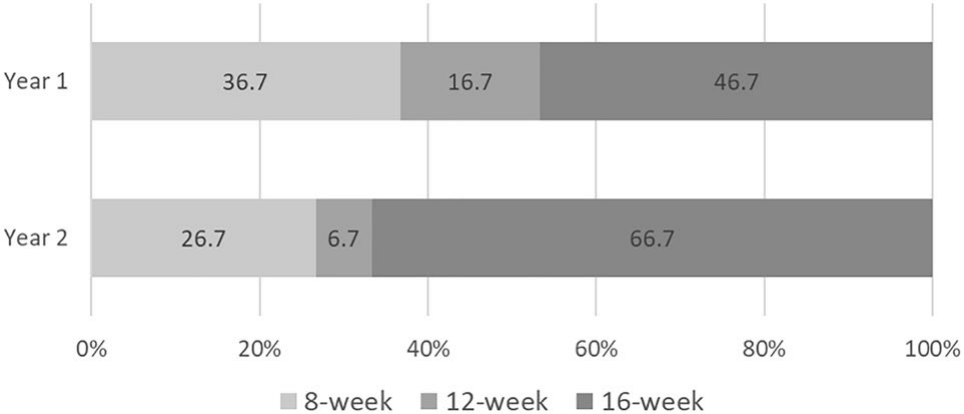
Distribution of various treatment intervals (8 weeks, 12 weeks, and 16 weeks) after 1 and 2 years.

In the FAS population (n = 40), the mean number of IAIs through 1 year and 2 years was 6.6 ± 1.5 and 10.1 ± 3.3, respectively. In the PPS population (n = 30), the mean number of IAIs through 1 year and 2 years was 7.0 ± 1.1 and 11.4 ± 2.1, respectively. In this population, 8-week intervals were used in 36.7% (11/30) and 26.7% (8/30) of patients at the end of the 1 and 2 years, respectively. Twelve-week treatment intervals were used in 16.7% (5/30) and 6.7% (2/30) of patients at the end of 1 and 2 years, respectively. Sixteen-week treatment intervals were used in 46.7% (14/30) and 66.7% (20/30) of patients at the end of 1 and 2 years, respectively (Fig. 4).

### Additional outcomes

In the FAS population (n = 40), the mean largest ETDRS BCVA gain from baseline over the 2-year period was 11.1 ± 9.4 letters, which was achieved at 46.1 ± 34.1 weeks. In the 33 eyes that met the VISTA and VIVID criteria, the mean largest ETDRS BCVA gain from baseline over the 2-year period was 12.2 ± 9.9 letters, which was achieved at 45.0 ± 32.4 weeks. Similarly, in the PPS population (n = 30), the mean largest ETDRS BCVA gain from baseline over the 2-year period was 12.4 ± 10.3 letters, which was achieved at 54.8 ± 32.7 weeks. In the 23 eyes that met the VISTA and VIVID criteria, the mean largest ETDRS BCVA gain from baseline over the 2-year period was 14.5 ± 10.8 letters, which was achieved at 55.8 ± 29.7 weeks. In the FAS population, the mean maximum CST decrease over the 2-year period was 226.4 ± 114.6 µm, which was achieved at 50.1 ± 37.4 weeks. In the 33 eyes that met the VISTA and VIVID criteria, the mean maximum CST decrease was 227.6 ± 122.5 µm, which was achieved at 48.6 ± 37.6 weeks. Similarly, in the PPS population, the mean maximum CST decrease over the 2-year period was 246.2 ± 114.3 µm, which was achieved at 61.9 ± 35.7 weeks. In the 23 eyes that met the VISTA and VIVID criteria, the mean maximum CST decrease was 254.1 ± 124.5 µm, which was achieved at 63.3 ± 35.4 weeks.

### Adverse events

Ocular adverse events included eye pain (2/40, 5%), progression of cataract (2/40, 5%), itchy eyes (1/40, 2.5%), conjunctival hemorrhage (1/40, 2.5%), increased intraocular pressure (IOP) (1/40, 2.5%), watering of the eye (1/40, 2.5%), and blepharitis (1/40, 2.5%), which were generally consistent with those of other intravitreal anti-VEGF agents and typical of those seen with intravitreal injections^6,11,25^. Topical IOP-lowering medications were administered to the patient with raised IOP; subsequently, IOP became normal. No additional treatment was needed in other cases. Serious ocular adverse events included central retinal occlusion without evidence in 1 patient (2.5%), which suggested a direct relationship with IAI. No cases of endophthalmitis were reported in previous studies with the use of anti-VEGF agents^25,26^. Serious systemic events included pneumonia (1/40, 2.5%), cellulitis of the leg (1/40, 2.5%), and cerebral infarction (1/40, 2.5%) without evidence, which suggested a direct relationship with IAI. These patients were managed by the respective specialists and were excluded from the study. All serious adverse events were reported to the institutional review board and study sponsor over the 2-year duration of the trial; the board approved the reporting without comment.

## Discussion

The current study is 1 of the first to prospectively report the efficacy of a T&E regimen with aflibercept that set the longest treatment interval to 16 weeks for DME. In the present FAS population, a mean VA gain of 5.0 letters and CST reduction of 164.1 µm with an average of 10.1 IAIs were observed. In the PPS population who completed 2 years of follow-up, a mean VA gain of 6.1 letters and CST reduction of 184.1 µm, with an average of 11.4 IAI, was observed.

The excellent therapeutic effects of anti-VEGF agents in DME and gaining of vision are widely accepted^3–6,11,12,14,27^. However, the high socioeconomic burden of this therapy hinders its widespread application in patients with DME because anti-VEGF treatments span several years^7,28,29^. As an approach to solve this problem, some groups reported a good therapeutic efficacy of the T&E regimen which set the IAI interval to a maximum of 12 weeks in DME^21,22^. The ALTAIR study that examined a T&E regimen with aflibercept for AMD reported that up to week 96, the injection interval was extended to 16 weeks for 46.3% patients in the group with 4-week adjustments^23^. Therefore, we hypothesized that T&E regimens with aflibercept may extend the injection intervals to longer than 12 weeks, which should further reduce the number of injections. Consequently, we performed the present prospective study to investigate the efficacy of aflibercept in the T&E regimen with maximum treatment interval of 16 weeks. As many as 73.3% of patients with DME achieved IAI intervals ≥ 12 weeks (12 weeks: 6.7%; 16 weeks: 66.7%) at the end of the second year as anticipated. In the phase III trial of aflibercept for DME—the VIVID study that included Japanese patients (76/403, 18.9%)^11^—the number of IAIs was 22.6 ± 5.8 in the monthly-injection arm and 13.6 ± 2.9 in the bimonthly-injection arm^6,11^. Although direct comparisons are not possible between the current small single-arm study and a big, randomized control trial (VIVID), the number of IAIs in patients with similar baseline visual acuity (73 ≥ baseline BCVA > 24 letters) was 9.7 ± 3.4 (Supplementary Table S3). These results suggest that the T&E regimen can potentially reduce the required number of IAIs for DME compared with the bimonthly fixed-dosing regimen, especially, in the second year.

When we aim to avoid over-treatment that might be associated with fixed-dosing regimen and adjust the number of IAIs based on the T&E regimen, we have to maintain the therapeutic effects in terms of visual and anatomical outcomes. Dugel et al.^30^ investigated the correlation between the baseline and final visual acuities following anti-VEGF therapy across large, randomized control trials. They reported that there usually is a strong inverse correlation between the mean baseline VA and VA gain at 12 months of anti-VEGF treatment. They reported that lower baseline VA strongly predicts better VA gain in patients with DME. Furthermore, diabetic retinopathy is a variable disease that is dependent on both local and systemic factors, which are very difficult to manage. Consequently, they concluded cross-trial comparisons based on the changes in BCVA. They mentioned that such comparisons should be performed cautiously and only after adjusting for baseline BCVA. Although the baseline BCVA was > 24 letters in all participants in this study, it was > 73 letters in some participants. Since the baseline BCVA of VIVID/VISTA studies was 24–73 letters, we selected 23 participants with baseline BCVA of 24–73 letters for comparisons. The mean VA gain in this sub-group was 7.8 ± 12.5, while it was 9.4 ± 10.5 in the bimonthly IAI arm in the VIVID study. Anatomically, in these 23 participants, the mean CST decreased by 189.5 ± 124.6 µm, which was comparable to those of VIVID (195.8 ± 141.7 µm) and VISTA (191.1 ± 160.7 µm) studies. These data suggest that the T&E regimen of aflibercept with adjunct focal/grid laser to diminish extra-foveal leakage required fewer IAI treatments over 2 years than the conventional fixed-dosing regimen while producing comparable morphological and functional therapeutic efficacy.

However, 26.7% participants still required 8-week intervals to achieve resolution even after the second year. We previously reported that eyes with leaking foveal microaneurysms at baseline require a greater number of anti-VEGF injections than those without such leaks^14,15^. In this study, although no significant correlation between foveal leaking microaneurysms at baseline and number of IAIs was observed (Spearman’s ρ = 0.08405; 95% confidence interval: − 0.3018 to 0.4462; P = 0.6647) (Supplementary Fig. S1a) a highly significant statistical positive correlation was observed between foveal leaking microaneurysms at 24 weeks and number of IAIs (Spearman’s ρ = 0.7476; 95% confidence interval: 0.5167–0.8772; P < 0.001) (Supplementary Fig. S1B). These results suggest that the eyes with foveal leaking microaneurysms at 24 weeks after the induction phase required a greater number of IAIs.

As indicated in Fig. 2, the magnitude of VA changes from baseline varies between patients. Therefore, early indicators of long-term vision outcomes are of interest to both the treating physicians and patients. Previous reports have demonstrated that eyes with relatively large early improvements achieve a greater long-term VA gain than do eyes that do not achieve substantial early gains^31,32^. This trend was also confirmed in the present study, where the eyes that gained a median of ≥ 4 letters at 12 weeks after 3 initial IVIs (induction phase) achieved greater improvements in vision (13.8 ± 9.5 letters) than the remaining eyes (− 4.3 ± 9.2 letters) at 1 year (P < 0.001). The former group demonstrated greater average vision gain than the latter group (9.8 ± 13.2 vs. 2.0 ± 6.5 letters, respectively) at 2 years, although the difference was not statistically significant probably because the standard deviation increased (Supplementary Fig. S2).

According to the results of the present study, the following 3 new findings can be explained to patients with DME to motivate them to continue anti-VEGF therapy. First, more than 70% of patients with a center-involving DME treated by a T&E regimen of aflibercept and adjunct focal/grid laser achieved IAI intervals ≥ 12 weeks (12 weeks: 6.7%; 16 weeks: 66.7%) at the end of 2 years. Second, patients with foveal leaking microaneurysms at 24 weeks may require more IAIs even after the second year. This finding may help patients to comply with fixed-dosing anti-VEGF injections. We may also be able to suggest other treatment strategies, such as changing to other anti-VEGF agents, steroids, and vitrectomy for such patients. Third, patients with early improvements in vision following the induction phase tended to achieve better final improvements in vision, which was comparable to those observed in previous randomized clinical trials.

Several limitations of this study should be acknowledged. First, this was an open-label, single-arm study with a relatively small sample size. Additionally, direct comparisons could not be made between the efficacies of the T&E regimen with aflibercept and other aflibercept dosing strategies, such as fixed treatment and PRN dosing. However, this has been supplemented with comparisons to the VIVID study by adjusting for the baseline BCVA. Second, this study only included Japanese participants. Yamashiro et al.^33^ have reported that among AMD-susceptibility genes, rs10490924 in ARMS2/HTRA1 was significantly associated with an additional anti-VEGF treatment requirement. In VISTA and VIVID, which were used for comparison with the results of the current study, more than 80% of the participants were Caucasian and less than 20% were Asian^11^. The differences in responses to anti-VEGF therapy in DME among races are not clear, however, the racial differences may have affected the results. Future studies are needed to determine the responses to anti-VEGF therapy in DME for each race. Third, the patients enrolled in the current study showed relatively well-controlled diabetes. This study used the same criteria as the VISTA and VIVID studies and excluded poorly controlled diabetes mellitus patients in order to compare the results more accurately. As a result, the mean HbA1c of the enrolled patients was 7.2 ± 1.0%, which was relatively low, while the mean HbA1c was 7.7 ± 1.8% in a study of real-world DME treatment in Japan reported by Shimura et al.^34^. This discrepancy could have affected the results. Thus, to make future surveys more clinically relevant, it will be necessary to include patients with poorly controlled diabetes in the cohort. Finally, we set the IAI interval to a maximum of 16 weeks. Therefore, this study did not verify the extent to which the IAI interval can be extended; further studies are required to evaluate the same. However, the strengths of the current study include the prospective and multicenter design. Additionally, all data were collected by a contract research organization, which ensured accuracy and fairness of data. In most studies, the timing of laser treatment is entirely at the discretion of the treating physicians. Although, by requiring it to be performed 1 week after the first IAI in all patients, we believe that we were able to fairly evaluate the effects of IAIs using a T&E regimen. We have previously reported that concomitant use of focal/grid photocoagulation with intravitreal ranibizumab injection in patients with DME allows for fewer intravitreal ranibizumab injections while maintaining comparable positive therapeutic effects^14,15^. Therefore, our use of focal/grid photocoagulation may have contributed to the reduction in the number of IAIs required. As mentioned earlier, this study is a single-arm study and therefore this cannot be stated definitively. In the future, it will be necessary to investigate the therapeutic effect of a T&E regimen using IAI alone without laser treatment.

In conclusion, a T&E aflibercept regimen with the longest treatment interval set to 16 weeks, with adjunct focal/grid laser, may be a rational 2-year treatment strategy for DME.

## Supplementary Information

Supplementary Information.

## Data Availability

The datasets generated during and/or analyzed during the current study are available from the corresponding author on reasonable request.

## References

[1] Ghanchi F, Hazel CA (2016). South Asian diabetic macular oedema treated with ranibizumab (ADMOR)-real-life experience. Eye (Lond)..

[2] Treatment techniques and clinical guidelines for photocoagulation of diabetic macular edema. Early Treatment Diabetic Retinopathy Study Report Number 2. Early Treatment Diabetic Retinopathy Study Research Group. *Ophthalmology*. **94,** 761–774 (1987). 10.1016/s0161-6420(87)33527-4 3658348

[3] Diabetic Retinopathy Clinical Research Network *et al*. Randomized trial evaluating ranibizumab plus prompt or deferred laser or triamcinolone plus prompt laser for diabetic macular edema. *Ophthalmology*. **117,** 1064–1077.e35 (2010). 10.1016/j.ophtha.2010.02.031 PMC2937272 20427088

[4] Arevalo JF (2009). Primary intravitreal bevacizumab for diffuse diabetic macular edema: The Pan-American Collaborative Retina Study Group at 24 months. Ophthalmology.

[5] Brown DM (2013). Long-term outcomes of ranibizumab therapy for diabetic macular edema: The 36-month results from two phase III trials: RISE and RIDE. Ophthalmology.

[6] Heier JS (2016). Intravitreal aflibercept for diabetic macular edema: 148-week results from the VISTA and VIVID studies. Ophthalmology.

[7] Maniadakis N, Konstantakopoulou E (2019). Cost effectiveness of treatments for diabetic retinopathy: A systematic literature review. Pharmacoeconomics..

[8] Jaffe DH, Chan W, Bezlyak V, Skelly A (2018). The economic and humanistic burden of patients in receipt of current available therapies for nAMD. J. Comp. Eff. Res..

[9] Shimura M (2019). Real-world management of treatment-naive diabetic macular oedema in Japan: Two-year visual outcomes with and without anti-VEGF therapy in the STREAT-DME study. Br. J. Ophthalmol..

[10] Nguyen QD (2012). Ranibizumab for diabetic macular edema: Results from 2 phase III randomized trials: RISE and RIDE. Ophthalmology.

[11] Brown DM (2015). Intravitreal aflibercept for diabetic macular edema: 100-week results from the VISTA and VIVID studies. Ophthalmology.

[12] Do DV (2012). One-year outcomes of the da Vinci Study of VEGF Trap-Eye in eyes with diabetic macular edema. Ophthalmology.

[13] Glassman AR (2020). Five-year outcomes after initial aflibercept, bevacizumab, or ranibizumab treatment for diabetic macular edema (Protocol T Extension Study). Ophthalmology.

[14] Hirano T (2019). Effect of leaking foveal microaneurysms on the treatment of center-involving diabetic macular edema: A pilot study. Ophthalmic. Res..

[15] Hirano T (2017). Effect of leaking perifoveal microaneurysms on resolution of diabetic macular edema treated by combination therapy using anti-vascular endothelial growth factor and short pulse focal/grid laser photocoagulation. Jpn. J. Ophthalmol..

[16] Neto HO (2017). Multicenter, randomized clinical trial to assess the effectiveness of intravitreal injections of bevacizumab, triamcinolone, or their combination in the treatment of diabetic macular edema. Ophthalmic Surg. Lasers Imaging Retina..

[17] Freund KB (2015). Treat-and-extend regimens with anti-VEGF agents in retinal diseases: A literature review and consensus recommendations. Retina..

[18] Chin-Yee D, Eck T, Fowler S, Hardi A, Apte RS (2016). A systematic review of as needed versus treat and extend ranibizumab or bevacizumab treatment regimens for neovascular age-related macular degeneration. Br. J. Ophthalmol..

[19] Haga A, Kawaji T, Ideta R, Inomata Y, Tanihara H (2018). Treat-and-extend versus every-other-month regimens with aflibercept in age-related macular degeneration. Acta Ophthalmol..

[20] Payne JF (2019). Randomized trial of treat and extend ranibizumab with and without navigated laser versus monthly dosing for diabetic macular edema: TREX-DME 2-year outcomes. Am. J. Ophthalmol..

[21] Pak KY (2020). One-year results of treatment of diabetic macular edema with aflibercept using the treat-and-extend dosing regimen: The VIBIM Study. Ophthalmologica..

[22] Curry BA, Sanfilippo PG, Chan S, Hewitt AW, Verma N (2020). Clinical outcomes of a treat and extend regimen with intravitreal aflibercept injections in patients with diabetic macular edema: Experience in clinical practice. Ophthalmol. Ther..

[23] Ohji M (2020). Efficacy and safety of intravitreal aflibercept treat-and-extend regimens in exudative age-related macular degeneration: 52- and 96-week findings from ALTAIR: A randomized controlled trial. Adv. Ther..

[24] Park SJ (2016). Intraocular pharmacokinetics of intravitreal aflibercept (eylea) in a rabbit model. Investig. Ophthalmol. Vis. Sci..

[25] Do DV (2011). The DA VINCI Study: Phase 2 primary results of VEGF Trap-Eye in patients with diabetic macular edema. Ophthalmology.

[26] DeCroos FC (2017). Treat-and-extend therapy using aflibercept for neovascular age-related macular degeneration: A prospective clinical trial. Am. J. Ophthalmol..

[27] Elman MJ (2015). Intravitreal ranibizumab for diabetic macular edema with prompt versus deferred laser treatment: 5-year randomized trial results. Ophthalmology.

[28] Muston D (2018). An efficacy comparison of anti-vascular growth factor agents and laser photocoagulation in diabetic macular edema: A network meta-analysis incorporating individual patient-level data. BMC Ophthalmol..

[29] Terasaki H (2019). Efficacy and safety outcomes of intravitreal aflibercept focusing on patients with diabetic macular edema from Japan. Retina..

[30] Dugel PU (2016). Baseline visual acuity strongly predicts visual acuity gain in patients with diabetic macular edema following anti-vascular endothelial growth factor treatment across trials. Clin. Ophthalmol..

[31] Pieramici D (2018). Outcomes of diabetic macular edema eyes with limited early response in the VISTA and VIVID studies. Ophthalmol. Retina..

[32] Bressler NM (2018). Early response to anti-vascular endothelial growth factor and two-year outcomes among eyes with diabetic macular edema in Protocol T. Am. J. Ophthalmol..

[33] Yamashiro K (2017). A prospective multicenter study on genome wide associations to ranibizumab treatment outcome for age-related macular degeneration. Sci. Rep..

[34] Shimura M (2020). Real-world management of treatment-naïve diabetic macular oedema in Japan: Two-year visual outcomes with and without anti-VEGF therapy in the STREAT-DME study. Br. J. Ophthalmol..

