# Supplementary material for: Outcomes of a 2-year treat-and-extend regimen with aflibercept for diabetic macular edema
Source: Sci Rep. 2021 Feb 24;11:4488. doi: 10.1038/s41598-021-83811-y (PMC7904904; doi:10.1038/s41598-021-83811-y)
Supplement: Supplementary file 1 — Supplementary Information. [file 41598_2021_83811_MOESM1_ESM.pdf]

Supplemental Data for:

Outcomes of a 2-year treat-and-extend regimen with aflibercept for diabetic macular edema

Takao Hirano[1]*, Yuichi Toriyama[1], Yoshihiro Takamura[2], Masahiko Sugimoto[3], Taiji Nagaoka[4], Yoshimi Sugiura[5], Fumiki Okamoto[5], Michiyuki Saito[6], Kousuke Noda[6], Shigeo Yoshida[7], Akihiro Ishibazawa[8], Osamu Sawada[9], Toshinori Murata[1]

[1] Department of Ophthalmology, Shinshu University School of Medicine, 3-1-1 Asahi, Matsumoto, Nagano 390-8621, Japan
[2] Department of Ophthalmology, Faculty of Medical Sciences, University of Fukui, Eiheiji, Yoshida, Fukui 910-1193, Japan
[3] Department of Ophthalmology, Mie University Graduate School of Medicine, 2-174, Edobashi, Tsu, Mie 514-8507, Japan
[4] Department of Ophthalmology, Nihon University Itabashi Hospital, 30-1 Ooyaguchikami-machi, Itabashi, Tokyo 173-8610, JAPAN
[5] Department of Ophthalmology, University of Tsukuba Faculty of Medicine, 2-1-1 Amakubo, Tsukuba, Ibaraki 305-8576, Japan
[6] Department of Ophthalmology, Faculty of Medicine and Graduate School of Medicine, Hokkaido University, Kita 15, Nishi 7, Kita, Sapporo 060-8638, Japan
[7] Department of Ophthalmology, Kurume University School of Medicine, 67 Asahi, Kurume, Fukuoka 830-0011, Japan
[8] Department of Ophthalmology, Asahikawa Medical University, 2-1-1 Midorigaokahigasi Asahikawa 078-8510, Japan
[9] Department of Ophthalmology, Shiga University of Medical Science, Seta Tsukinowa, Otsu, Shiga 520-2192, Japan

Supplemental Figure Captions
Supplemental Figures 1, 2

Supplemental Table Captions
Supplemental Tables 1-3

Supplemental Figure Captions

Supplemental Figure 1. Correlation between foveal leaking microaneurysms (MAs) at baseline (A) and at 24 weeks (B) and the number of intravitreal aflibercept injections (IAI).

Supplemental Figure 2. Association between an early response to intravitreal aflibercept injections (IAIs) and the 2-year visual acuity outcomes in all per-protocol set of participants (n=30). Change in the best-corrected visual acuity (BCVA) from baseline according to early response at 12 weeks. *P < 0.05 vs. eyes with less BCVA improvement (post-hoc multiple comparisons using Bonferroni method). SD: standard deviation. Missing values were imputed using the “last observation carried forward” method.

Supplemental Figure 1.

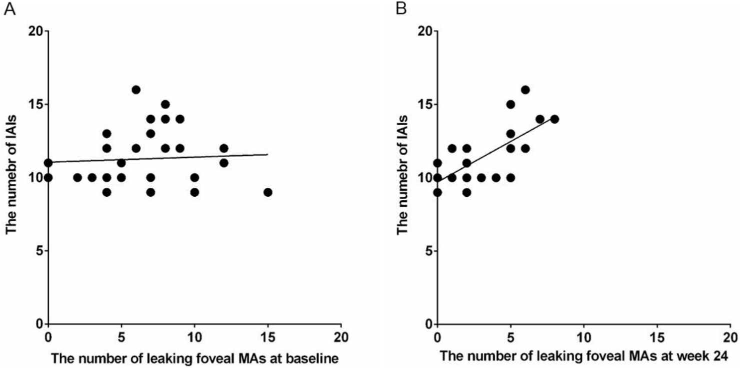

Supplemental Figure 2.

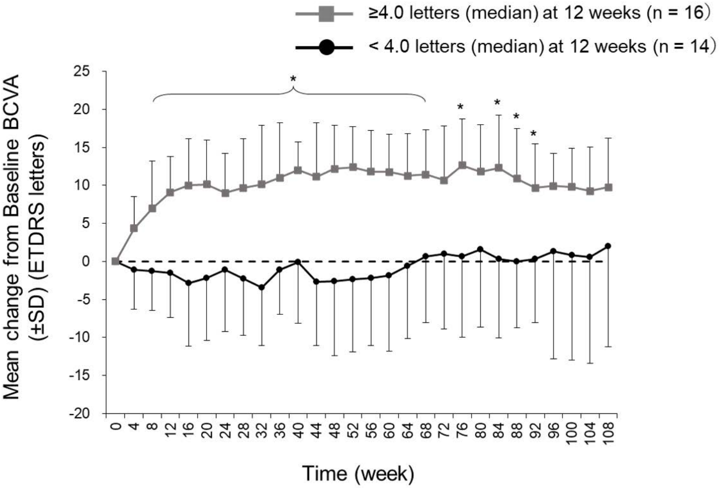

Supplemental Table 1. Eligibility criteria

Inclusion Criteria

[1] Patients with type 1 or type 2 diabetes mellitus 20 years of age or older

[2] Patients with fovea-involving diabetic macular edema (judged with central subfield thickness on OCT)

[3] Patients with reduced visual acuity associated with diabetic macular edema

[4] Patients whose best corrected visual acuity is ≥20/320 (≥24 letters) by the ETDRS visual acuity test. (In the VIVID and VISTA studies, the upper limit of patient's visual acuity in the ETDRS visual acuity test was set at ≤20/40 [≤73 letters]. In this study, however, no upper limit is set.)

[5] Patients with a central subfield thickness (CST) of 300 μm or more on the OCT retinal map (measured in mm) as an objective finding

[6] Patients who are willing and able to visit the study site, as scheduled, and undergo scheduled examinations in this study

[7] Patients who can provide written voluntary consent to participate in this study after receiving adequate explanation

Exclusion Criteria

Patients who meet none of the following exclusion criteria will be enrolled in this study.

- The condition of the fellow eye is worse than that of the study eye.
- A history of vitreous surgery, including encircling buckling
- Any retinal photocoagulation treatment within 90 days before the scheduled

date of the first injection of aflibercept

- Two or more sessions of direct laser or Grid laser treatment
- Topical corticosteroid treatment (intravitreal injection or subtenon injection) within 120 days before the scheduled date of the first injection of aflibercept
- Treatment with injection of any anti-VEGF agent into the study eye within 90 days before the scheduled date of the first injection of aflibercept
- Highly active, proliferative diabetic retinopathy in the study eye
- A history of idiopathic or autoimmune uveitis
- A history of cataract surgery in the study eye within the previous 90 days
- Aphakic eyes
- A history of posterior capsulotomy in the study eye within the previous 30 days
- A history of any intraocular surgery in the study eye within the previous 90 days
- The presence of vitreomacular traction is confirmed along with reduced visual acuity that is not attributable to edema
- Iris neovascularization, vitreous hemorrhage, or tractional retinal detachment in the study eye
- Preretinal fibrosis in the study eye
- It is considered difficult to improve visual acuity due to morphological changes, such as atrophy of the macula
- A history of endophthalmitis in the study eye
- Blepharitis, keratitis, scleritis, or conjunctivitis in both eyes at the time of start of the study (baseline)

- Glaucoma with poor intraocular pressure (IOP) control, a history of glaucoma filtering surgery, or scheduled filtering surgery in the study eye
- IOP of the study eye: ≥25 mmHg
- Myopia of -8D or greater
- A history of any disease that may cause reduced visual acuity other than DME (for example, retinal vascular occlusion, retinal detachment, macular hole, and macular angiogenesis of any cause)
- Patients with only one eye (study eye)
- Optic media in which it is difficult to obtain fundus photographs or OCT images
- Severe systemic infections at baseline
- Systemic anticoagulation therapy within 180 days before the scheduled date of the first injection of aflibercept
- Poorly controlled diabetes mellitus (HbA1c >12.0% as a rough measure)
- Poorly controlled hypertension (systolic blood pressure ≥160 mmHg or diastolic blood pressure ≥95 mmHg as a rough measure)
- A history of cerebrovascular accident and/or myocardial infarction within 180 days before the scheduled date of the first injection of aflibercept
- Patients on dialysis and patients with renal failure requiring dialysis
- Systemic disease requiring medications that may affect the results of this study
- Pregnant or lactating women
- Women who want to have a baby and do not want to use any contraceptive methods during the study period

- A history of allergy to fluorescein
- A history of hypersensitivity to aflibercept
- Participation in another clinical study within 30 days before the scheduled date of the first injection of aflibercept

Patients who are judged by the investigator to be ineligible as a subject for any other reason

Supplemental Table 2. Patient demographics and baseline characteristics of patients in the protocol set

Characteristic	Participants (n = 40)
Age, years	65.1 (10.0)
Female, n (%)	12 (40.0)
HbA1c, %	7.4 (1.0)
Duration of diabetes, years	12.1 (8.7)
Central retinal thickness, ㎛	499.2 (105.6)
Previous treatment for DME, n<br>(direct or grid laser, anti-VEGF, naïve)	9/7/17
DME morphological subtypes, n<br>(DRT alone, DRT+CME, DRT+SRD, DRT+CME+SRD)	2/19/4/5
DR severity, n<br>(mild/moderate/severe NPDR/PDR)	2/15/9/4
Cr, mg/dl	0.89 (0.32)
Hb, g/dl	13.6 (1.6)
Blood pressure, mmHg	
systolic	132.4 (14.7)
diastolic	77.0 (11.1)

Data are presented as mean (standard deviation)

CME: cystoid macular edema; Cr: creatinine; DME: diabetic macular edema; DR: diabetic retinopathy; DRT: diffused retinal thickening; Hb: Hemoglobin; HbA1c: Hemoglobin A1c; NPDR: non-proliferative diabetic retinopathy; PDR: proliferative diabetic retinopathy; SRD: serous retinal detachment; VEGF: vascular endothelial growth factor.

Supplemental Table 3. Comparison of our study with the VISTA and VIVID studies

Study Arms	Mean number of IAI	Mean improvement in BCVA (letters)	Mean reductions in CST (um)
**Our study**			
All FAS participants	10.1±3.3	5.0±12.1	164.1±119.4
FAS participants with 73 ≥ baseline BCVA > 24 letters	9.7±3.4	5.9±12.8	163.7±120.6
All PPS participants	11.4±2.1	6.1±11.8	184.1±122.3
PPS participants with 73 ≥ baseline BCVA > 24 letters	11.7±2.8	7.8±12.5	189.5±124.6
**VISTA Study**			
2q4	21.3±5.8	11.5±13.8	191.4±180.0
2q8	13.5±2.9	11.1±10.7	191.1±160.7
**VIVID Study**			
2q4	22.6±5.8	11.4±11.2	211.8±150.9
2q8	13.6±2.9	9.4±10.5	195.8±141.7

BCVA: best corrected visual acuity; CST: central macular thickness; 2q4: Intravitreal 2 mg aflibercept injection (IAI) every 4 weeks; 2q8: IAI every 8 weeks after three initial monthly doses
